# The clinicopathological features of colorectal mucinous adenocarcinoma and a therapeutic strategy for the disease

**DOI:** 10.1186/1477-7819-10-109

**Published:** 2012-06-15

**Authors:** Masakatsu Numata, Manabu Shiozawa, Takuo Watanabe, Hiroshi Tamagawa, Naoto Yamamoto, Soichiro Morinaga, Kazuteru Watanabe, Teni Godai, Takashi Oshima, Shoichi Fujii, Chikara Kunisaki, Yasushi Rino, Munetaka Masuda, Makoto Akaike

**Affiliations:** 1 Department of Gastroenterological Surgery, Kanagawa Cancer Center, 1-1-2 Nakao, Asahi-ku, Yokohama, Kanagawa, 241-0815, Japan; 2 Gastroenterological Center, Yokohama City University Medical Center, 4-57 Urafune-cho, Minami-ku, Yokohama, Kanagawa, 232-0024, Japan; 3 Department of Surgery, Yokohama City University, 3-9 Fukuura, Kanazawa-ku, Yokohama, Kanagawa, 236-0004, Japan

**Keywords:** Mucinous adenocarcinoma, Colorectal cancer, Clinicopathological feature

## Abstract

**Background:**

The guidelines established by the National Comprehensive Cancer Network do not describe mucinous histology as a clinical factor that should influence the therapeutic algorithm. However, previous studies show conflicting results regarding the prognosis of colorectal mucinous adenocarcinoma. In this study, we described the clinicopathological features of mucinous adenocarcinoma in Japan, to identify optimal therapeutic strategies.

**Methods:**

144 patients with mucinous and 2673 with non-mucinous adenocarcinomas who underwent primary resection in two major centers in Yokohama, Japan were retrospectively evaluated for clinicopathological features and treatment factors. A multivariate analysis for overall survival followed by the comparison of overall survival using Cox proportional hazard model were performed.

**Results:**

Patients with mucinous adenocarcinoma had larger primary lesions, higher preoperative CEA levels, a deeper depth of invasion, higher rates of nodal and distant metastasis, and more metastatic sites. A multivariate analysis for overall survival revealed a mucinous histology to be an independent prognostic factor. In the subgroup analysis stratified by stage, Patients diagnosed as StageIII and IV disease had a worse survival in mucinous adenocarcinoma than non-mucinous, while survival did not differ significantly in patients diagnosed as Stage0-II disease. In StageIII, local recurrence in rectal cases and peritoneal dissemination were more frequently observed in patients with a mucinous histology.

**Conclusions:**

Our study indentified that mucinous adenocarcinoma was associated with a worse survival compared with non-mucinous in patients with StageIII and IV disease. In rectal StageIII disease with mucinous histology, additional therapy to control local recurrence followed by surgical resection may be a strategical alternative. Further molecular investigations considering genetic features of mucinous histology will lead to drug development and better management of peritoneal metastasis

## Background

Colorectal cancer is the third most common cancer and the fourth most frequent cause of cancer death worldwide [1]. Mucinous adenocarcinoma (MA) is diagnosed when more than 50% of the tumor comprises a mucinous pattern upon histological examination [2]. MA makes up 6 to 20% of all colorectal cancers [3-8], and differs from non-mucinous adenocarcinoma (NMA) with regard to its clinicopathological characteristics, distinct genetic profiles, and pathogenic pathways [9-12].

The prognostic significance of MA is controversial. In previous studies, mucinous histology was reported not to be an independent prognostic factor for survival [13,14]. The guidelines established by the National Comprehensive Cancer Network (NCCN) do not describe mucinous histology as a clinical factor that should influence the therapeutic algorithm [15,16]. However, in some studies, it is reported that MA is associated with worse clinicopathological characteristics [17-19] and a poorer prognosis than NMA [5,17,20-23].

The lack of a consensus may be the result of the low ratio of MA to all colorectal cancers and the limited detection power to clarify the differences between MA and NMA.

We conducted a retrospective analysis of patients with colorectal cancer at two major centers to identify the clinicopathological features of MA, and also investigated the recurrence to establish an optimal therapeutic strategy for MA.

## Methods

### Patients

The data from 2,817 patients with colorectal cancer in two major centers, Kanagawa Cancer Center and Yokohama City University Medical Center, between 2001 and 2010 were investigated. Written informed consent was obtained from the patient for publication of this report and any accompanying images. All patients initially underwent resection of a primary lesion followed by adjuvant chemotherapy when diagnosed as stage III disease. In tumor, node metastases (TNM) stage T3 to T4, lower-rectal cases, total mesorectal excision (TME) and lateral node dissection were routinely performed at initial resection. No patients were treated with neo- or adjuvant radiation therapy.

The analyzed patients were diagnosed with MA, defined as tumors with more than 50% of the tumor volume comprising mucin, or with NMA, defined as tumors without any mucinous features, or with a less than 50% mucinous component [2]. Patients diagnosed with signet ring cell carcinoma, undifferentiated carcinoma, and other histological types were excluded from the analysis.

The covariates included in the study were gender, age, location of the tumor, size of the primary tumor, preoperative serum carcinoembryonic antigen (CEA) level, depth of invasion, lymph node metastasis, distant metastasis, operating facility, and histological type. The pathological tumor status was coded using the TNM classification system [24]. The use of adjuvant chemotherapy for patients with curative resection, as well as additional therapy (chemotherapy and/or surgery) for recurrent and non-curative cases were also recorded.

In the analysis of the survival rates, all cases were divided into 3 groups, that is, patients without any metastases (stage 0 to II [24]), patients with regional lymph node metastasis but without distant metastasis (stage III), and patients with distant metastasis (stage IV).

### Statistical analysis

The two groups of patients (MA and NMA) were compared using 2 × 2 tables for binary factors using the χ^2^-test, or Fisher’s exact test where appropriate. Overall survival was calculated from the date of surgery for the primary lesion until death from any cause, or was censored at the last follow-up visit. Survival data were analyzed using the Kaplan-Meier method. A comparison of survival curves was carried out using the log-rank test. The prognostic significance was analyzed by multivariate Cox proportional hazard models. *P*-values < 0.05 were considered statistically significant, and all *P*-values correspond to two-sided significance tests.

## Results

Of the 2,817 colorectal cancer patients, MA accounted for 5.1% (144) of the colorectal cancer cases. The distribution of the patients’ characteristics is shown in Table 1. The distribution for gender, age, and location of MA was similar to that of NMA. The patients with MA had significantly larger primary lesions, higher preoperative serum CEA levels, deeper invasion, higher nodal and distant metastasis rates, and a larger number of metastatic sites compared to the patients with NMA.

**Table 1:** Comparison of clinicopathological characteristics in non-mucinous and mucinous adenocarcinoma

**Characteristics**		**NMA**	**MA**	***P*-value**
		(n = 2,673)	(n = 144)	
Gender				0.772
	Female	1,072	56	
	Male	1,601	88	
Age, years				0.271
	< 65	1,183	57	
	≧ 65	1,490	87	
Location				0.090
	Colon	1,516	92	
	Rectum	1,157	57	
Size, cm				< 0.001
	< 5	1,715	59	
	≥ 5	958	85	
Preoperative serum CEA, ng/ml				< 0.001
	<5.0	1,858	80	
	≧5.0	815	64	
Depth of invasion^a^				< 0.001
	T1, T2	979	13	
	T3, T4	1,694	131	
Lymph node metastasis^a^				0.002
	N0	1,602	68	
	N1, N2	1,071	76	
Distant metastasis^a^				0.010
	M0	2,319	114	
	M1	354	30	
Number of metastatic sites				0.019
	0	2,319	114	
	1	269	25	
	≧ 2	86	5	

^a^Staging of tumor (T), nodes (N), and metastases (M) [24]; *NMA*, non-mucinous adenocarcinoma; *MA*, mucinous adenocarcinoma.

Table 2 shows the distribution of treatment factors, including the curability of the first surgery, rate of adjuvant chemotherapy, chemotherapy for advanced or recurrent cases, and additional surgery for liver and lung metastasis. For these factors, there were no significant differences between the MA and NMA groups. Unlike western countries, neo- or adjuvant radiation therapy for patients with stage II and III disease is not commonly performed in Japan.

**Table 2:** Comparison of treatment factors in non-mucinous and mucinous adenocarcinoma

**Treatment factors**	**NMA**	**MA**	***P*-value**
Curability of first surgery	n = 2,673	n = 144	0.063
Complete	2,411	123	
Incomplete	262	21	
Curability of first surgery in rectal cancer	n = 1,157	n = 57	0.885
Complete	1,101	54	
Incomplete	56	3	
Lateral node dissection in rectal cancer	n = 1157	n = 57	0.104
Yes	189	14	
No	968	43	
Adjuvant chemotherapy	n = 773	n = 54	0.192
Yes	357	20	
No	416	34	
Chemotherapy for advanced/recurrent cases	n = 639	n = 55	0.597
Yes	220	17	
No	419	38	
Additional resection			
Liver resection for metastatic cases	n = 249	n = 11	0.884
Yes	73	3	
No	176	8	
Lung resection for metastatic cases	n = 123	n = 5	0.179
Yes	33	0	
No	90	5	

*NMA*, non-mucinous adenocarcinoma; *MA*, mucinous adenocarcinoma.

**Figure 1:**
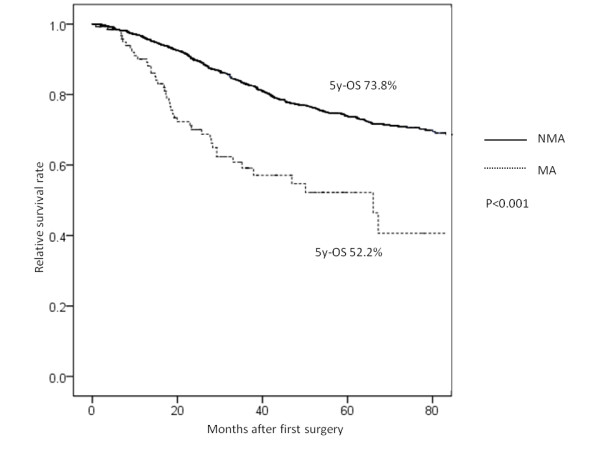
**The 5-year relative survival rate of patients with mucinous adenocarcinoma (52.2%) was significantly worse than those with non-mucinous adenocarcinoma (73.8%) as determined by the log-rank test (*P*** **< 0.001).** 5yOS, 5-year overall survival.

To clarify the prognostic factors for colorectal cancer, univariate and multivariate analysis were carried out. Mucinous histology was noted to be one of the independent prognostic factors for overall survival in univariate and multivariate analysis (Tables 3 and 4). The 5-year relative survival rate of the MA patients was 52.2%, which was significantly worse than that of the NMA patients (73.8%), with a median follow-up of 52 months (range 1 to 128 months) (Figure 1).

**Table 3:** Univariate analysis of overall survival in colorectal adenocarcinoma

**Variables**		**HR (95% CI)**	***P*-value**
Gender			0.004
	Female	1.0	
	Male	1.349 (1.098, 1.656)	
Age, years			0.049
	< 65	1.0	
	≧ 65	1.217 (1.001, 1.480)	
Location			0.049
	Colon	1.0	
	Rectum	1.212 (1.000, 1.468)	
Size, cm			< 0.001
	< 5	1.0	
	≧ 5m	2.432 (2.001, 2.955)	
Preoperative serum CEA, ng/ml			< 0.001
	< 5.0	1.0	
	≧ 5.0	3.223 (2.654, 3.915)	
Depth of invasion^a^			< 0.001
	T1, T2	1.0	
	T3, T4	6.122 (4.311, 8.694)	
Lymph node metastasis^a^			< 0.001
	N0	1.0	
	N1, N2	4.527 (3.972, 5.122)	
Distant metastasis^a^			< 0.001
	M0	1.0	
	M1	8.133 (6.697, 9.877)	
Operating facility			< 0.001
	Center A	1.0	
	Center B	2.365 (1.943, 2.878)	
Histological type			< 0.001
	NMA	1.0	
	MA	2.614 (1.905, 3.585)	

^a^Staging of tumor (T), nodes (N), and metastases (M) [24]; *HR*, hazard ratio; *CI*, confidence interval; *NMA*, non-mucinous adenocarcinoma; *MA*, mucinous adenocarcinoma.

**Table 4:** Multivariate Cox proportional hazards analysis of overall survival in colorectal adenocarcinoma

**Variables**		**HR (95% CI)**	***P*-value**
Gender			0.020
	Female	1.0	
	Male	1.278 (1.040, 1.572)	
Age, years			0.007
	< 65	1.0	
	≧ 65	1.317 (1.078, 1.608)	
Location			0.020
	Colon	1.0	
	Rectum	1.267 (1.038, 1.547)	
Size, cm			0.112
	< 5	1.0	
	≧ 5	1.184 (0.961, 1.459)	
Preoperative serum CEA, ng/ml			< 0.001
	< 5.0	1.0	
	≧ 5.0	1.483 (1.203, 1.828)	
Depth of invasion^a^			< 0.001
	T1, T2	1.0	
	T3, T4	2.253 (1.534, 3.308)	
Lymph node metastasis^a^			< 0.001
	N0	1.0	
	N1, N2	2.427 (1.893, 3.105)	
Distant metastasis^a^			< 0.001
	M0	1.0	
	M1	4.165 (3.350, 5.179)	
Operating facility			0.032
	Center A	1.0	
	Center B	1.192 (1.002, 1.395)	
Histological type			< 0.001
	NMA	1.0	
	MA	2.226 (1.618, 3.062)	

^a^Staging of tumor (T), nodes (N), and metastases (M) [24]; *HR*, hazard ratio; *CI*, confidence interval; *NMA*, non-mucinous adenocarcinoma; *MA*, mucinous adenocarcinoma.

**Figure 2:**
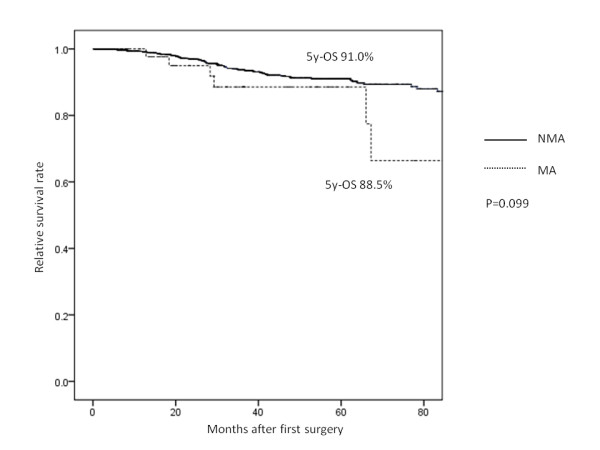
The 5-year overall survival of patients with stage I or stage II non-mucinous adenocarcinoma and mucinous adenocarcinoma.

**Figure 3:**
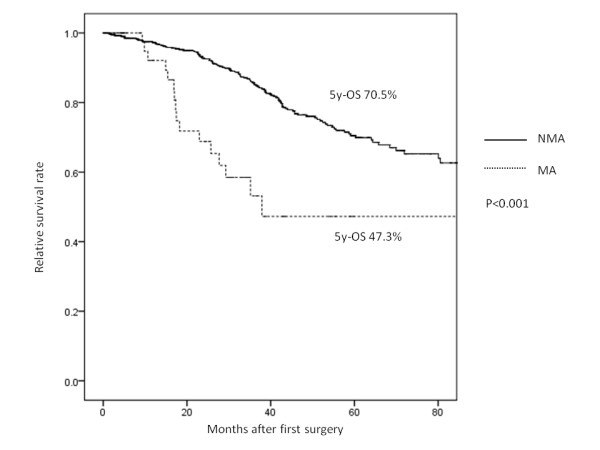
The 5-year overall survival of patients with stage III non-mucinous adenocarcinoma and mucinous adenocarcinoma.

**Figure 4:**
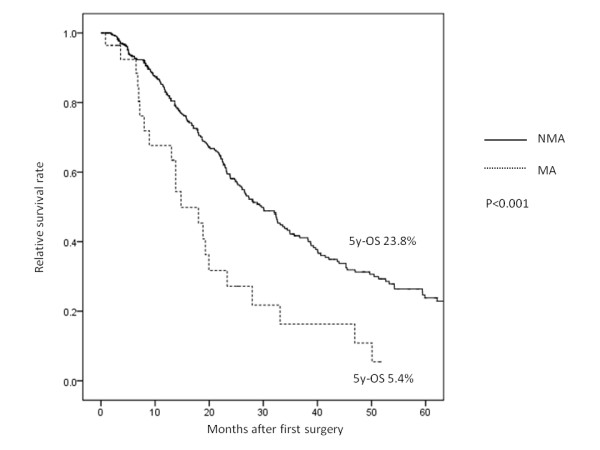
The 5-year overall survival of patients with stage IV non-mucinous adenocarcinoma and mucinous adenocarcinoma.

We next divided all cases into 3 groups (stage 0 to II, stage III, and stage IV) to compare the overall survival in each group. There were no significant differences in 5-year survival in stage 0 to II based on the histological status (MA: 88.5% vs. NMA: 91.0%, *P* = 0.099) (Figure 2). However, in stage III (MA: 47.3% vs. NMA: 70.5%, *P* < 0.001) (Figure 3) and stage IV (MA: 5.4% vs. NMA: 23.8%, *P* < 0.001) (Figure 4), the MA patients had a significantly worse survival rate than the NMA patients.

The recurrence pattern in stage III was analyzed (Table 5). The number of patients with any recurrence in the MA and NMA groups was 17 (31.4%) and 182 (23.5%), respectively. The rate of liver metastasis, distant lymph node metastasis, lung metastasis, and other site recurrence was similar between the two groups. Peritoneal dissemination (7.4% vs. 2.3%, *P* = 0.049), and local recurrence (9.2% vs. 2.3%, *P* = 0.013) were more frequently observed in the MA group. All of the locally recurrent cases in the MA group were rectal cases.

**Table 5:** The comparison of recurrent pattern in patients with stage III disease

**Recurrent pattern**	**NMA**	**MA**	***P*-value**
	(n = 774)	(n = 54)	
Liver metastasis	80 (10.3%)	4 (7.4%)	0.643
Peritoneal metastasis	18 (2.3%)	4 (7.4%)	0.049
Distant lymph node metastasis	30 (3.8%)	1 (1.8%)	0.715
Lung metastasis	60 (7.7%)	4 (7.4%)	1.000
Local recurrence	18 (2.3%)	5 (9.2%)	0.013
Other site recurrence	16 (2.0%)	3 (5.5%)	0.121

*NMA*, non-mucinous adenocarcinoma; *MA*, mucinous adenocarcinoma.

The treatment factors were compared in stage IV disease (Table 6). However, the curability of the first resection, first regimen of chemotherapy, and the rate of additional resection did not differ significantly between the MA and NMA groups.

**Table 6:** Comparison of treatment factors in patients with stage IV disease

**Treatment factors**	**NMA**	**MA**	**P-value**
	(n = 349)	(n = 28)	
Curability of first surgery			0.353
Complete	96 (27.5%)	10 (35.7%)	
Incomplete	253 (72.5%)	18 (64.3%)	
Regimen of first chemotherapy			0.362
OXA-based	25 (7.1%)	3 (10.7%)	
IRI-based	18 (5.1%)	3 (10.7%)	
5-FU intravenously	43 (12.3%)	4 (14.2%)	
5-FU orally	36 (10.3%)	4 (14.2%)	
Additional resection			
Liver resection	75 (21.4%)	3 (10.7%)	0.228
Lung resection	10 (2.8%)	0 (0.0%)	1.000

*OXA*, oxaliplatin; *IRI*, irinotecan; *5-FU*, 5-fluolouracil; *NMA*, non-mucinous adenocarcinoma; *MA*, mucinous adenocarcinoma.

## Discussion

In this study, the data from 2,817 colorectal cancer patients who underwent surgery in two major medical centers in Yokohama city, Japan, were analyzed. In these 2,817 patients, the incidence of MA was 5.1%, which generally corresponds to the rate in other Asian countries, as reported by Chew (6.0% from Singapore) [3] and Safaee (8.5% from Iran) [6]. Other studies from Western countries described that the proportion of MA ranged from 11 to 20% [4,5,7,8], which is higher than the rate for Asian countries. This disparity may reflect the differences in geographic, ethnic and dietary factors.

In the analysis of the patients’ characteristics, the MA group had worse clinical factors, including larger primary lesions, deeper invasion, higher rates of nodal and distant metastasis, and a larger number of metastatic sites compared to the NMA group. The previous reports showed that younger patients, larger tumors, higher rates of lymph node metastasis, and peritoneal metastasis were correlated with MA histology when compared to NMA histology [17-19], which was mostly in agreement with our results.

The reason why MA patients have worse characteristics than NMA patients is not fully understood. Sugarbaker *et al*. demonstrated that the more malignant characteristics of MA may be partly due to the production of mucus under pressure, which allows the MA cells to gain access to the peritoneal cavity. Moreover, the fluid produced by MA is taken up by the lymphatic system, which might help to promote tumor spread into regional lymph nodes [25].

It remains unclear whether MA adversely affects survival in colorectal cancer patients. In some studies, MA has been shown to be a significant prognostic factor [5,17,20-23], while others have found no such evidence [13,14,26]. Both the American Joint Committee on Cancer and the College of American Pathologists consider that the MA subtype has not been proven to be a statistically significant prognostic factor [14,27]. The contradictions in the various studies may be explained by the geographical and racial variations in the epidemiology of colorectal cancer [28,29], disparities in the criteria for defining MA [22,30], and insufficient sample sizes to disclose any differences. In this study, we compared the relative survival rate of MA and NMA patients in a relatively large number of patients, and revealed that the MA patients had significantly worse survival than the NMA patients. Furthermore, multivariate analysis demonstrated that MA histology is an independent prognostic factor.

Based on our findings, we also performed an investigation to establish an optimal therapeutic strategy for MA. To exclude the bias caused by stage, which is related to the status of lymph node metastasis and distant metastasis, we divided all cases into three subgroups and analyzed the relative survival rate in each group. Consequently, the prognosis of patients with MA was similar to that of patients with NMA in stage I/II, whereas it was significantly worse for MA patients in both stage III and stage IV. Some of the above-mentioned literature supports the relatively worse survival of MA patients when the subjects are limited to stage III and stage IV disease [8,18,21], which is consistent with our results. According to these results, we speculated that the worse survival of stage III and IV may contribute to the relatively poor overall prognosis of the MA cases.

In the analysis of the recurrence pattern of stage III disease, the incidence of local recurrence and peritoneal metastasis was significantly higher in patients with MA than in those with NMA. In addition, all cases of local recurrence in the MA group were in patients with rectal disease. Differences in the rates of liver, lung, and distant lymph node metastases were not statistically significant.

In stage IV disease, we compared the treatment factors, including the curability of the first resection, first chemotherapeutic regimen, and the need for additional resection (liver and lung resection). This analysis revealed that equivalent treatments were performed for both MA and NMA patients.

Our results indicate that better management of the local and peritoneal recurrence of stage III disease may improve the survival of MA patients. In terms of local control, previous studies have shown that the difference in survival between MA and NMA patients was mainly related to tumors with a rectal location [18], because MA cases are more likely to be locally recurrent [31], which supports our present results. Green *et al*. pointed out that the lymphatic drainage of the pelvis is more extensive and varied compared with that of the colon [5].

In the NCCN guidelines for treatment of rectal cancer [16], it is recommended to perform pre- or postoperative radiation therapy for patients with T3 to T4, or N1 to N2 disease. However, In Japan, Sugihara *et al*. showed that the five-year overall survival and the five-year locally controlling ratio in patients with T3 to T4 rectal cancer treated by TME plus lateral node dissection, was 79.7% and 92.0% respectively [32]. Consequently, TME and lateral node dissection is considered a standard therapy for T3 to T4 rectal cancer, and neo- or adjuvant radiation therapy is not commonly selected in Japan.

Considering our result, the application of pre- or postoperative radiation therapy [33-36] may be a strategy to prevent the development of local recurrence in MA cases.

We also found that peritoneal metastasis was a significant site of recurrence for MA in stage III disease. Metastasis to the peritoneum is regarded to be a fatal manifestation of gastrointestinal cancer, and is associated with a median survival time of 5.2 to 12.6 months [37,38]. In terms of the sensitivity to chemotherapy, previous reports have demonstrated that a mucinous histology generally predicts a reduced response to a 5-FU-, oxaliplatin-, and irinotecan-based regimen [39,40].

It is well known that there are several molecular pathways involving oncogene (for example *KRAS*) and the suppressor gene in colorectal carcinogenesis [41], and cetuximab is an established drug used for downstream blocking of the EGFR-KRAS pathway. However, according to Hanski *et al*., MA histology is characterized by a high frequency of *KRAS* mutations, and a high frequency of microsatellite instability [9], suggesting that MA histology generally has drug-resistant properties of cetuximab. It is also revealed that MA has different molecular alterations and genetic subtypes to NMA [42]. Detailed molecular and genetic analyses to detect specific pathways of MA will help to develop new systemic chemotherapy, which is necessary to improve peritoneal metastasis and overall survival in patients with MA.

## Conclusion

Our study indentified MA histology as an independent prognostic factor, and revealed that MA was associated with worse survival compared with NMA in patients with stage III and IV disease. The ability of MA to disseminate and infiltrate more aggressively than NMA appears to be responsible, at least in part, for the higher rate of failure in stage III and IV, which are the main reasons for the overall poorer prognosis of patients with MA. Controlling local recurrence and managing peritoneal metastasis are necessary to improve the overall survival in patients with MA.

Besides considering radiation therapy after TME and radical lymph node dissection for locally advanced rectal cases, further investigations focusing on the genetic and molecular characteristics of MA, will help better management of MA histology.

## Abbreviations

CEA: Carcinoembryonic antigen; 5-FU: 5-fluolouracil; IRI: Irinotecan; MA: Mucinous adenocarcinoma; NCCN: National Comprehensive Cancer Network NMA, non-mucinous adenocarcinoma; OXA: Oxaliplatin; TME: Total mesorectal excision; TNM: Tumor, node, metastases.

## Competing interests

There are no financial or non-financial competing interests to declare in relation to this manuscript.

## Authors’ contributions

MN carried out the analysis of data and wrote the manuscript. MS, TW, HT, NY, SM, KW, TG, TO, SF, CK, NY, YR, MM, and MA made the database of patients. All authors read and approved the final manuscript.
